# Quantitative bioimage analytics enables measurement of targeted cellular stress response induced by celastrol-loaded nanoparticles

**DOI:** 10.1007/s12192-019-00999-9

**Published:** 2019-05-11

**Authors:** Erik Niemelä, Diti Desai, Emine Lundsten, Jessica M. Rosenholm, Pasi Kankaanpää, John E. Eriksson

**Affiliations:** 1 Cell Biology, Faculty of Science and Engineering, Åbo Akademi University, Turku, Finland; 2 Turku Bioscience Centre, University of Turku and Åbo Akademi University, Turku, Finland; 3 Pharmaceutical Sciences Laboratory, Faculty of Science and Engineering, Åbo Akademi University, Turku, Finland

**Keywords:** Image quantification, BioImageXD, Nuclear stress bodies, Mesoporous silica nanoparticles, Targeted drug delivery, Celastrol

## Abstract

The cellular stress response, which provides protection against proteotoxic stresses, is characterized by the activation of heat shock factor 1 and the formation of nuclear stress bodies (nSBs). In this study, we developed a computerized method to quantify the formation and size distribution of nSBs, as stress response induction is of interest in cancer research, neurodegenerative diseases, and in other pathophysiological processes. We employed an advanced bioimaging and analytics workflow to enable quantitative detailed subcellular analysis of cell populations even down to single-cell level. This type of detailed analysis requires automated single cell analysis to allow for detection of both size and distribution of nSBs. For specific induction of nSB we used mesoporous silica nanoparticles (MSNs) loaded with celastrol, a plant-derived triterpene with the ability to activate the stress response. To enable specific targeting, we employed folic acid functionalized nanoparticles, which yields targeting to folate receptor expressing cancer cells. In this way, we could assess the ability to quantitatively detect directed and spatio-temporal nSB induction using 2D and 3D confocal imaging. Our results demonstrate successful implementation of an imaging and analytics workflow based on a freely available, general-purpose software platform, BioImageXD, also compatible with other imaging modalities due to full 3D/4D and high-throughput batch processing support. The developed quantitative imaging analytics workflow opens possibilities for detailed stress response examination in cell populations, with significant potential in the analysis of targeted drug delivery systems related to cell stress and other cytoprotective cellular processes.

**Electronic supplementary material:**

The online version of this article (10.1007/s12192-019-00999-9) contains supplementary material, which is available to authorized users.

## Introduction

The cellular stress response is an example of a specific cellular process that has received broad attention in research related to aging, cancer, neurodegenerative diseases, and other pathophysiological processes. The stress response is characterized by the formation of nuclear stress bodies (nSBs), which are characteristically formed in primate cells upon proteotoxic stress, such as heat shock, oxidative stress, many pharmacological agents, heavy metals, proteasome inhibitors, and amino acid analogues (Sarge et al. 1993; Holmberg et al. 2000; Jolly et al. 2002). In this study, we wanted to analyze targeted induction of nSBs by advanced imaging combined with suitable software tools to transform the image data produced into meaningful information (Cardona and Tomancak 2012; Carpenter et al. 2012; Kankaanpää et al. 2012; Myers 2012). Bioimaging has grown to become one of the key methodologies in biomedical research and while there are nowadays numerous suitable software packages available, developing tools and workflows for different and specific purposes is still often necessary in constantly evolving research scenarios (Upla et al. 2004; Karjalainen et al. 2008; Sukumaran et al. 2012; Kankaanpää et al. 2012; Myers 2012).

Heat shock factor 1 (HSF1) is a transcription factor that activates the expression of many target genes, and in the case of classical heat shock response (HSR), HSF1 increases the expression of molecular chaperones called heat shock proteins (Hsps) that facilitate protein folding and maintenance of a functional proteostasis during stressed conditions (Cotto et al. 1997; Alastalo et al. 2003; Sandqvist et al. 2009; Biamonti and Vourc'h 2010). A unique feature of the primate heat shock response is that HSF1 not only initiates transcription of its target Hsp genes, but also binds and accumulates to specific 9q12 loci at repetitive Satellite III sequences where HSF1 is responsible for transcribing noncoding Satellite III RNAs (Biamonti and Vourc'h 2010). This accumulation of HSF1 can, in fact, be visualized with fluorescence microscopy as high-intensity spots inside the nucleus, with sizes between 0.3 and 3 μm, and this unique subnuclear structure is called nSBs (Holmberg et al. 2000). While counting and measuring nuclear stress bodies (nSBs) are of significant interest in many research situations, the quantification has been limited to manual counting (Cotto et al. 1997; Morimoto 1998; Jolly et al. 2002; Holmberg et al. 2000; Pirkkala et al. 2001; Alastalo et al. 2003; Sandqvist et al. 2009; Biamonti and Vourc'h 2010; Åkerfelt et al. 2010; Mendillo et al. 2012; Baron et al. 2013; Vihervaara et al. 2013). Manual quantification is laborious, subjective, and limited in terms of the parameters it can reliably produce, especially when estimating sizes of objects and distances between them (Carpenter et al. 2012; Cardona and Tomancak 2012; Kankaanpää et al. 2012; Myers 2012). We have now developed a computer-based workflow that enables quantification of the cell population positive for nSBs, as a tool for quantitative measurements on how different pharmacological and other stress treatments affect the cellular stress response machinery. Also, other parameters, such as size distribution and location of the nSBs, can easily be extracted with our method. Our solution is based on the combination of standard laser scanning confocal microscopy and a workflow on a large and versatile open source software platform for multi-dimensional bioimages, in this case BioImageXD. This software natively supports true 3D data in nearly all of its functionality, which includes flexible visualization and analysis tools, and batch processing that requires no programming or scripting (Kankaanpää et al. 2012). BioImageXD has been used in numerous applications in biomedical research, ranging from virology and nanoparticle internalization studies to cancer medicine and drug development (Upla et al. 2004; Karjalainen et al. 2008; Sukumaran et al. 2012; Kankaanpää et al. 2012).

Automated image analysis of nSBs in cell populations, including analysis on single cell level, opens significant possibilities for detailed analysis of HSF1 DNA-binding and transcription capabilities upon different proteotoxic stresses that are relevant for pathophysiology or pharmacological development. Protein aggregates are associated with many neurodegenerative diseases and systemic amyloidosis and, consequently, there has been significant pharmacological interest to follow activation of the heat shock response in specific cells by targeted drug delivery. In this respect, activation of HSF1 and thereby synthesis Hsps are of obvious interest as their induction will protect the cell from harmful protein aggregates by promoting renaturation of aggregated proteins back to their native conformation (Sarge et al. 1993; Holmberg et al. 2000; Jolly et al. 2002; Trott et al. 2008). Hence, an automated workflow to quantify nSB formation would be a significant asset in the field of proteostasis and proteotoxic pathophysiology (Jolly et al. 2002; Trott et al. 2008; Singh and Lillard 2009; Salminen et al. 2010; Baron et al. 2013; Vihervaara et al. 2013).

In order to have a pharmacologically relevant context, we utilized nanoparticles as targeted drug delivery systems. In this way, we had the possibility to evoke a specific response in a certain population of cells, while the rest of the cells remain unaffected (Singh and Lillard 2009; Rosenholm et al. 2009, 2010; Bae and Park 2011; Zwicke et al. 2012). In this study, we employed the poorly water-soluble drug celastrol as a model compound to study the cellular stress response and the formation of nSBs, as it has the ability to efficiently activate the cellular stress response (Westerheide et al. 2004, 2009; Niemelä et al. 2015). Target-selectivity was achieved by functionalizing the nanoparticle surface with folic acid (FA), as cells with high folate receptor (FR) expression can internalize such particles through receptor-mediated endocytosis (Leamon and Low 2001; Rosenholm et al. 2009; Kularatne and Low 2010). The majority of cancer cells express FR in significantly higher amounts than most normal cells, and this difference was utilized as a model system to evaluate the specificity of these celastrol-loaded FA-conjugated nanoparticles (Nakajima and Ikada 1995; Kennedy et al. 2003; Elnakat and Ratnam 2004; Parker et al. 2005; Rosenholm et al. 2006, 2008; Desai et al. 2014).

By using the imaging-based quantitation method, we could show that FA-conjugated nanoparticles were more efficiently endocytosed by FR-positive cells than FR-negative cells. Furthermore, the imaging-based quantification confirmed that the celastrol-loaded nanoparticles induced nSBs formation in FR-positive Hela cells and not in FR-negative A549 cells. Taken together, our results demonstrate that the developed automated detection workflow achieves detailed quantitative analysis of several facets of the heat shock response, as a specific cellular process, and that analysis was achieved both on population and single cell level. Because our work is based on a straightforward imaging modality and freely available open source software, our approach can easily be utilized and adapted by others working in the field of targeted drug delivery and cellular stress responses. Furthermore, the targeted induction of the stress response by celastrol has potential ramifications in diseases that are associated with protein aggregates and disturbed proteostasis.

## Materials and methods

### Cell culture and sample preparations

Hela human cervical carcinoma cells and A549 lung adenocarcinoma epithelial cells were cultured in Dulbecco’s Modified Eagle’s Medium (DMEM; Sigma-Aldrich, St. Louis, MO, USA) supplemented with 10% fetal calf serum (BioClear, Wiltshire, UK), 2 mM L-glutamin, 100 U/ml penicillin, 100 μg/ml streptomycin at 37 °C, 5% CO_2_, and 90% relative humidity. Heat shock treatments were performed as previously described at 42 °C in a constant temperature water bath (Cotto et al. 1997; Holmberg et al. 2000). The mesoporous silica nanoparticles (MSNs) were suspended in a HEPES buffer at neutral pH and ultra-sonicated for 30 min before administration.

### Detection of nuclear stress bodies by confocal microscopy

Hela and A549 cells were seeded on sterile coverslips that were mounted in 24 well cell culture dishes and grown either under control conditions at 37 °C or heat shocked at 42 °C for 15 min, 30 min, or 120 min, or then incubated with concentrations ranging from 0 to 60 μg/ml of MSNs for 3 h. For indirect immunofluorescence of the treated cells grown on the coverslips, samples were first washed with PBS containing 0.05% Tween 20, then either fixed and permeabilized for 6 min in − 20 °C methanol or with 3.7% paraformaldehyde containing 0.5% Triton X-100 for 15 min at room temperature. After three washes with PBS 0.05% Tween 20, cells were incubated for 1 h with blocking solution (10% fetal calf serum in PBS 0.05% Tween 20), then overnight with rabbit anti-HSF1 antibody (1:400 dilution; Holmberg et al. 2000). After three washes with PBS 0.05% Tween 20, samples were incubated for 1 h at room temperature with the secondary goat anti-rabbit antibodies in a dilution of 1:5000 (Alexa™ 546, Molecular Probes, Eugene, Oregon, USA). DNA was stained with VECTASHIELD® Mounting Media containing 4′, 6-diamidino-2-phenylindole dihydrochloride (DAPI; Vector Laboratories, Burlingame, California, USA). Images of cells were taken using a Zeiss LSM510-Meta Axiovert 200 M scanning confocal microscope (Carl Zeiss Microscopy GmbH, Jena, Germany) equipped with the SP2 software (version 3.2) and using a Plan-Apochromat 63x/1.4 Oil DIC objective. Using 405 nm excitation and 420–480 nm emission filtering for detecting the DAPI channel, and 543 nm excitation and > 560 nm emission filtering for the HSF1 channel, the two channels were separately recorded (multi-tracking). At least three representative images were acquired from three independent experiments for each sample. Imaging settings, such as laser powers, detector gains, pixel depth, and pixel density, were kept constant throughout all imaging, with pixel density set according to the Nyquist theorem. For 2D imaging, the focal plane was set to the middle of the cells, and for 3D imaging cells were entirely covered by optical sectioning.

The manual counting of nSBs and nSBs-positive cells is based solely upon visually inspecting 2D confocal images. First, the number of cells per image was counted from the DAPI channel, then from the HSF1 channel the number of nSBs was counted as high intensity spots inside the cell nucleus. For a cell to be classified as nSBs positive there had to be one or more nSBs inside the nucleus, from which it was possible to calculate the percentage of nSBs positive cells in the total population.

### Computer-based quantification of nuclear stress bodies

To quantify nSBs, local fluorescence intensity clusters of the HSF1 channel inside the cell nucleus were analyzed. First, cell nuclei were segmented in order to obtain the number of cells per image and to define the spatial region occupied by the nucleus in each cell, and for this purpose, the DAPI channel was used. The images were first filtered with Gaussian smooth (radius factor X: 15, Y: 15 Z: 1), and global threshold was used to divide the image into foreground and background (lower threshold 18, determined by visual evaluation). After that, object separation was used to identify individual nuclei and separate nuclei that were touching each other (segmentation level 10.0, remove objects with less voxels than 200). The BioImageXD Object separation is a multi-step process that uses, e.g., distance transform, morphological watershed segmentation and masking to create a label image from a binary image. It is able to separate objects touching each other, unlike regular labeling based on connectivity. The small object removal threshold in object separation was set to remove objects likely to be artifacts rather than cell nuclei. The resulting re-labeled images (segmented cell nuclei) were then analyzed to obtain the number of cells per image.

**Fig. 1:**
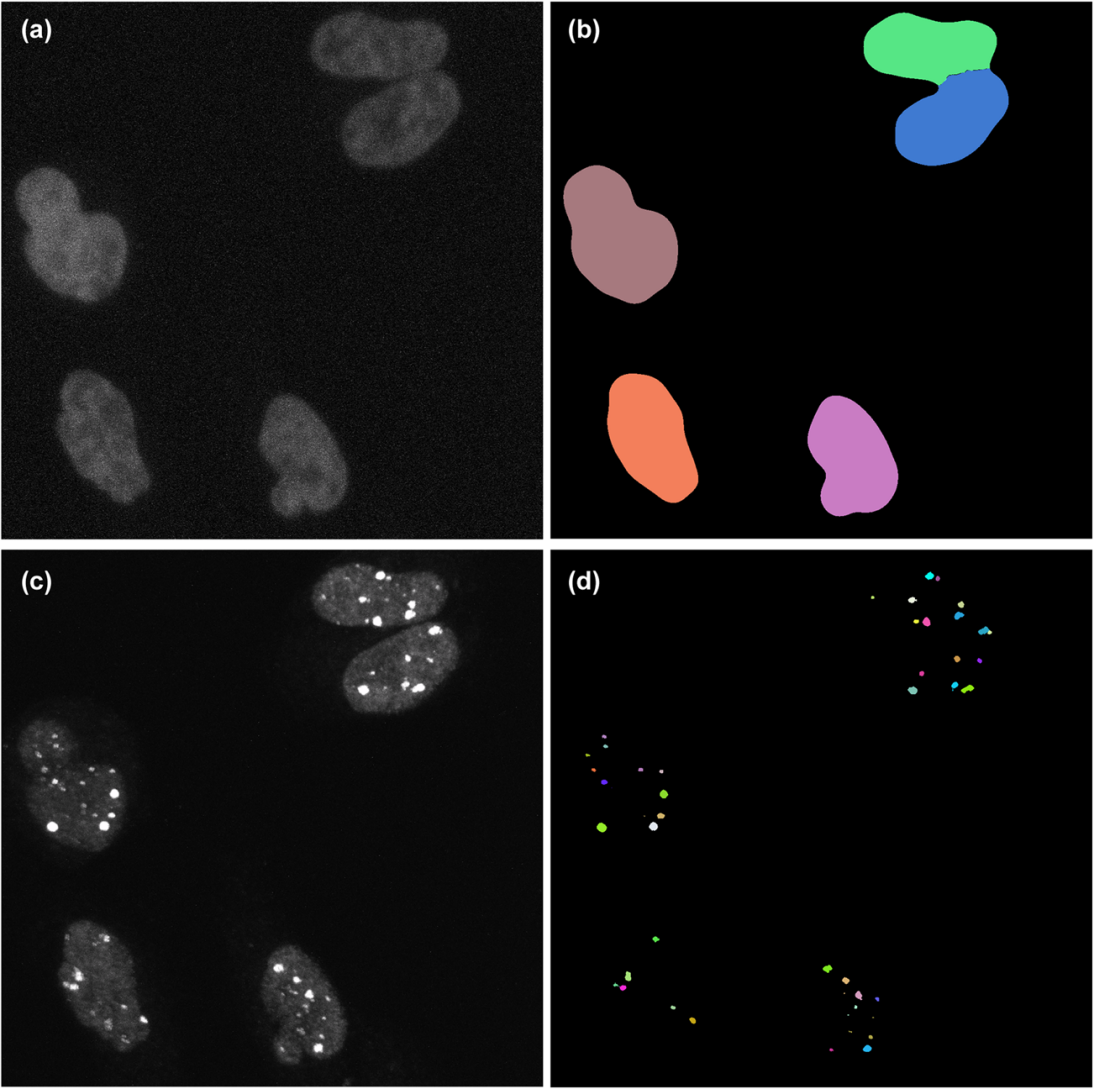
Quantification of nuclear stress bodies. The number of cells (*n*) per image was calculated from the DAPI channel **a** using global thresholding and object separation, resulting in each nucleus being represented by a separate object, with arbitrary coloring facilitating visual inspection **b**. The nSBs were segmented from the corresponding channel **c** by dynamic thresholding and object separation, for detecting local HSF1 intensity spots of varying intensity **d**. Object-based co-localization was used to analyze the overlapping nSBs objects with nucleus objects and cells with at least one stress granule in the nucleus were classified as stressed, images are maximum intensity projections of 3D datasets

Second, the nSBs were segmented from the HSF1 channel, in order to quantify nSBs and to classify cells into nSBs-positive and negative cells. The images were first mean-filtered (radius X: 5, Y: 5 and Z: 1), and by utilizing dynamic threshold with a radius of X:5, Y:5, and Z:1 together with threshold over statistics set on 16, the nSBs were separated from the background (Fig. 1c, d). Dynamic thresholding enabled the identification of nSBs that vary in intensity but are discernible in a local intensity environment. After the thresholding, BioImageXD object separation was used to identify individual nSBs and to separate nSBs touching each other (segmentation level 1.0). Objects (nSBs) with less voxels than 10 (corresponding to a 0.3-μm object diameter) were removed from the 3D images based on size distribution analysis, and for the high-throughput quantification based on 2D images a lower limit of 30 voxels (corresponding to 0.6 μm object diameter) was used.

Third, BioImageXD Object colocalization was employed to analyze the segmented nSBs and the segmented cell nuclei with respect to each other. Object colocalization analyzes the colocalization of two segmented channels (as opposed to conventional pixel/voxel-based colocalization analyses), and one of the many outputs of this procedure is that it can quantify the number of nSBs inside each cell’s nucleus, which in turn makes it possible to count the percentages of nSBs-positive cells within the sample population. In addition, each nSBs was quantitatively analyzed for object area. By utilizing the basic formula $d=2\ \sqrt{A}/\pi$, the diameter (d) of each nSBs was calculated from the area (A), producing quantifiable size distributions of the nSBs in the samples.

### Synthesis and characterization of mesoporous silica nanoparticles

The mesoporous silica nanoparticles were synthetized according to our previously published methods (Rosenholm et al. 2006, 2008, 2009). The synthesis was conducted under inert atmosphere conditions, where 1.152 ml of tetramethyl orthosilicate (TMOS) was mixed with 0.155 ml of APTMS (3-aminopropyl-trimethoxysilane) together with 2.5 mg fluorescein isothiocyanate (FITC), to create inherently fluorescent particles. The solution was mixed with an alkaline solution containing the structure-directing agent cetyltrimethyl ammonium chloride (CTACl) and methanol. The synthesis mixture used had a molar ratio of 0.9 TMOS: 0.1 APTMS: 1.27 CTACl: 0.26 NaOH: 1439 MeOH: 2560 H_2_O. The synthesis solution was stirred overnight at room temperature; thereafter, the particles were separated by centrifugation and washed with ethanol. The structure-directing agent was removed by ultrasonication in acidic (HCl) ethanol, centrifuged, and this process was repeated three times to further eliminate any residual surfactant. After that, the particles were washed with ethanol and vacuum-dried at room temperature overnight. Poly(ethylene imine), PEI, was grown onto the MSNs by hyperbranching surface polymerization of aziridine according to our previously described procedures (Rosenholm et al. 2006, 2008, 2009). After functionalization, the particles were washed repeatedly with ethanol and vacuum-dried at room temperature overnight. The amount of PEI-functionalization and celastrol loading degree was calculated on the basis of thermogravimetric analysis (TGA; Netzsch TG 209, Selb, Germany), using temperature intervals of 150 to 700 °C. PEI-functionalization was further confirmed by zeta potential measurements (Malvern ZetaSizer NanoZS, Malvern Instruments Ltd., Worcestershire, UK). Folic acid was covalently attached to the terminal amino groups of the PEI branches through carbodiimide coupling chemistry according to previous publications (Rosenholm et al. 2008, 2009, 2010). The hydrodynamic size of the particles, indicating the redispersibility in a HEPES buffer at pH 7.2, was confirmed by dynamic light scattering (DLS) measurements (Malvern ZetaSizer NanoZS, Malvern Instruments Ltd., Worcestershire, UK). In order to estimate the size, morphology, and monodispersity of these MSNs, scanning electron microscopy (SEM; Jeol JSM-6335F, Jeol Ltd., Tokyo, Japan) and transmission electron microscopy (TEM; JEM 1400-Plus, JEOL Ltd., Tokyo, Japan) were employed. The amount of folic acid conjugated onto the particle surface was calculated using UV-Vis spectroscopy.

### Assessment of particle endocytosis

Hela and A549 cells were seeded in 12 well culture dishes in 1 ml of cell medium for 24 h before the experiment. MSNs were suspended in cell medium at 1 μg/ml concentration and sonicated; thereafter, the medium containing particles or vehicle control (0.2% DMSO) was applied to 50–60% confluent cells and incubated for 3 h at 37 °C. Then, cells were trypsinized and harvested and the extracellular fluorescence was quenched by resuspension in trypan blue (200 μg/ml; Sigma-Aldrich, St. Louis, MO, USA) for 10 min at room temperature. The cells were washed and resuspended in phosphate buffer saline (PBS) and the amount of endocytosed particles inside cells was analyzed by BD FACSCalibur flow cytometer (FITC emission was detected with FL-1 channel; BD Pharmingen, San Diego, CA, USA). Then the data was analyzed with BD CellQuest Pro™ software (BD Pharmingen, San Diego, CA, USA) for acquiring percentages of FITC positive cells.

For validating particle endocytosis in folate receptor positive cells, confocal microscopy was employed on Hela cells that were grown on sterile coverslips and incubated with 10 μg/ml particles for 3 h. Extracellular fluorescence was quenched with trypan blue (200 μg/ml) for 10 min at room temperature. Cells were then washed with PBS and labeled with rhodamine-lectin (Vector Laboratories, Burlingame, California, USA) for 15 min at 37 °C. Thereafter, the cells were washed and fixed with 3.7% paraformaldehyde containing 0.5% Triton X-100 for 15 min at room temperature. The cells were washed and then mounted on glass slides using VECTASHIELD® mounting media containing DAPI (Vector Laboratories, Burlingame, California, USA). The particle endocytosis was imaged using a Zeiss LSM510 META confocal microscope (Carl Zeiss Microscopy GmbH, Jena, Germany) with a 63× oil objective, utilizing an excitation wavelength of 488 nm and a bandwidth emission filter with 500–550 nm for detecting the FITC channel.

### Induced heat shock measurement by Western blot

Celastrol-loaded MSNs ability to increase the Hsp70 protein levels in both cell lines were investigated by Western blot (WB). Hela and A549 cells were incubated with celastrol-loaded nanoparticles or empty particles for 6 h in order to obtain similar treatment conditions to that of the immunofluorescences images. Cells were thereafter lysed with laemmli buffer containing β-mercaptoethanol and samples were denaturated at 95 °C for 10 min. Ten micrograms of whole cell lysate was separated by 10% SDS-PAGE and the proteins were transferred to nitrocellulose membranes. Primary antibody Hsp70 (SPA-810; Stressgen Biotechnologies, California, USA) was used at a 1:10000 dilution and anti-β-actin antibody (Sigma-Aldrich, St. Louis, MO, USA) was used at a 1:1000 dilution as loading control. Secondary sheep anti-mouse antibody (GE healthcare, Buckinghamshire, UK) was used in a 1:50000 dilution, and the signal was detected using enhanced chemiluminescence (Amersham Biosciences Corp., Piscataway, NJ, USA).

### Statistical analysis

Statistical significance (*P* value) was determined by one-way analysis of variance with Bonferroni post hoc test, using GraphPad Prism® 5.0 (San Diego, California, USA). The error bars represent plus-minus the standard error of the mean (±SEM) of either number (*n*) of samples, cells or nSBs analyzed. Frequency distribution was used for representing the size distributions of nSBs from the quantified 3D images.

## Results

### Validation of the automated nuclear stress bodies quantification method

The 2D confocal images of the heat-treated samples were first quantified manually, and the results were then compared to the computer-based automated quantification using the same image datasets. For the computer-based counting of nSBs and nSBs-positive cells, multi-step workflows were developed in the BioImageXD software, version 1.0RC3 (Kankaanpää et al. 2012). In short, cell number and cell nucleus were detected from the DAPI channel and the nSBs were measured with dynamic thresholding and object separation from the HSF1 channel. Then by object-based co-localization, it was possible to calculate the percentages of the cell population that had one or more stress granule(s) in the nucleus (Fig. 1).

**Fig. 2:**
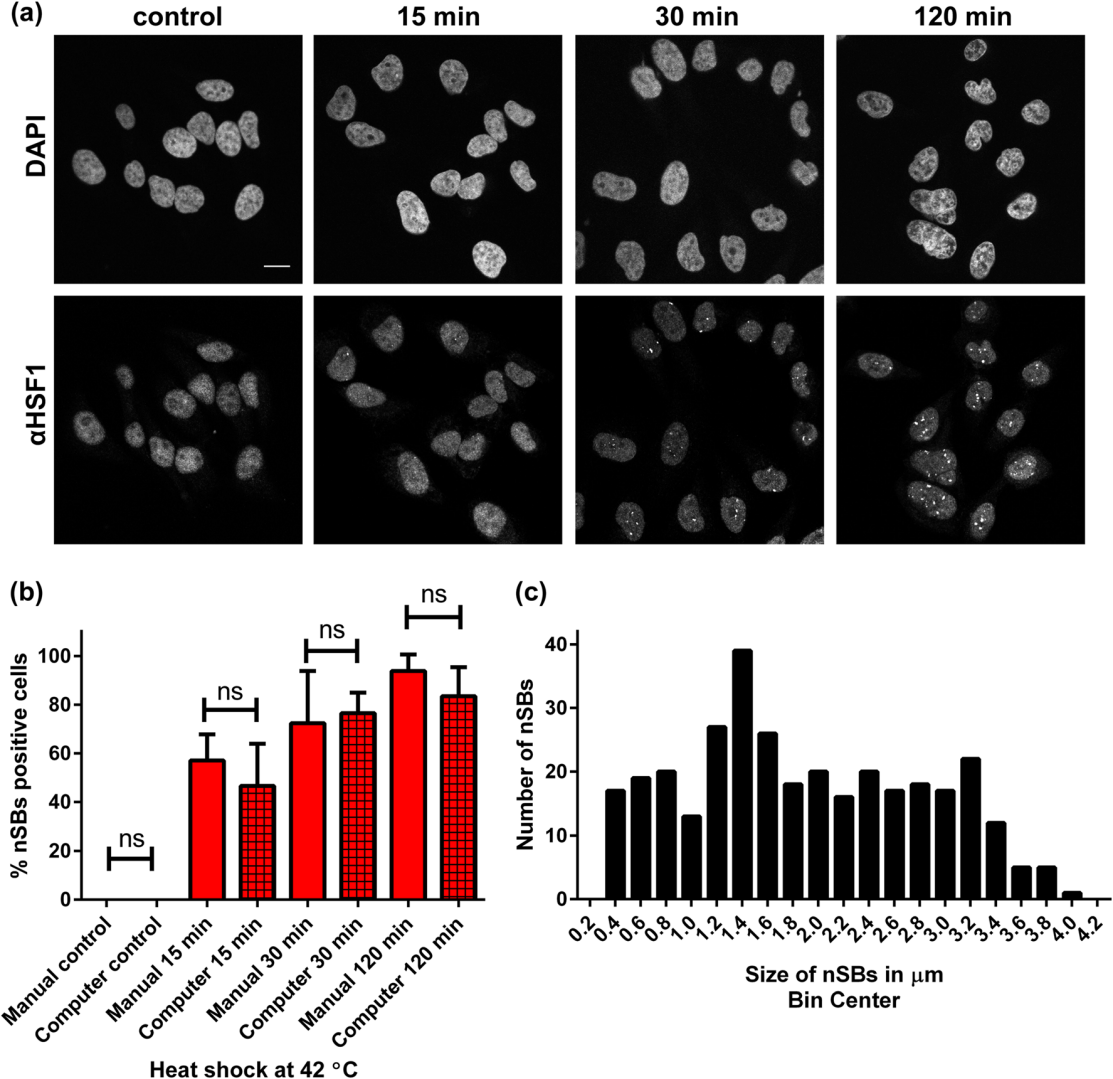
Formation kinetics of nSBs in heat shocked Hela cells. **a** The kinetics shows that cells that have been heat treated for 15 min at 42 °C only have a few HSF1 accumulations in the nucleus, whereas 30 and 120 min of heat shock induces clear formation of nSBs in almost all cells. Control cells that have been cultured in 37 °C does not form nSBs, scale bar 15 μm. **b** Both manual and computer-based quantification show similar heat shock induced nSBs kinetics, as there is no significant (ns) difference between the two methods. Manually analyzed cell number (*n* = 338), computer-based analyzed cell number (*n* = 357), error bars represents mean ± SEM. **c** Size distribution in μM of nSBs in Hela cells after 120 min of 42 °C heat shock, quantified from 3D datasets (*n* = 332)

The results from the heat-shocked samples show that both methods give similar mean values, and there is no statistical difference between the two quantifications, demonstrating the accuracy of the computer-based method (Fig. 2). In addition, both quantifications show that there is a temperature and time-dependent induction of nSBs in Hela cells that correlates with previous publications, further validating the usefulness of the computer-based method in measuring nSBs formation kinetics under different treatments (Fig. 2b; Cotto et al. 1997; Holmberg et al. 2000). More importantly by utilizing our automated workflow it was possible to acquire detailed information regarding the size distribution of the 3D dataset, which would have been laborious, subjective, and limited in terms of reproducibility if done manually (Fig. 2d). All quantitative analyses were carried out with BioImageXD built-in procedure lists, which enable setting up multi-step processing and analysis workflows as command pipelines with full parameter control, without the need for programming. Utilizing multiple procedure lists that can be run simultaneously for a large number of files with the BioImageXD batch processor, opens up the possibility for complex and high-throughput applications.

### Possibilities with 3D image analysis of nSBs

As BioImageXD has been designed to operate with 2D/3D and 4D data, all workflows presented in this paper are directly applicable to both 2D and 3D data. With 3D data, analyses are also automatically always conducted in 3D, not by repeating a 2D analysis for every “slice” of a 3D stack. In the context of nSBs, automated quantification that could open up numerous possibilities, as for instance accurate 3D size distributions of nSBs, could be measured without the need of programming. Here, we demonstrate this by analyzing true 3D size distribution of nSBs from 3D image datasets, acquired after 120 min of 42 °C heat-treated Hela cells. Such quantification would be very time consuming and nearly impossible to do accurately manually (Fig. 2c). The result of the size distribution shows that there is a slightly bimodal distribution, where most of the nSBs are in the size range of 1.4 μm; however, another distribution peak is seen in 3.2 μm size range. Interestingly the minimum nSBs size analyzed was exactly 0.373 μm and was therefore rounded up to the 0.4 μm size class and the maximum size was around 4.0 μm, taking together these results validates the already published observations of nSBs size distribution between 0.3 and 3 μm (Fig. 2c; Cotto et al. 1997; Morimoto 1998; Holmberg et al. 2000; Jolly et al. 2002). Such size distributions analysis can potentially be used to identify stress response kinetics of different treatments as the diameter and intensity of the nSBs correlates to the severity of the stress cells are subjected to (Cotto et al. 1997; Holmberg et al. 2000). While there is promising potential for the use of nSBs size distribution quantification, it should be noted that especially from 3D objects numerous other parameters can also be easily calculated. For instance, our protocols would enable quick quantification of the spatial distribution of nSBs within the nucleus, and shape analysis or volumes of different objects. While such parameters may not have been indicated to have any biological relevance in this paper, they might be worth investigating in the future.

### Celastrol induces the formation of nuclear stress bodies

**Fig. 3:**
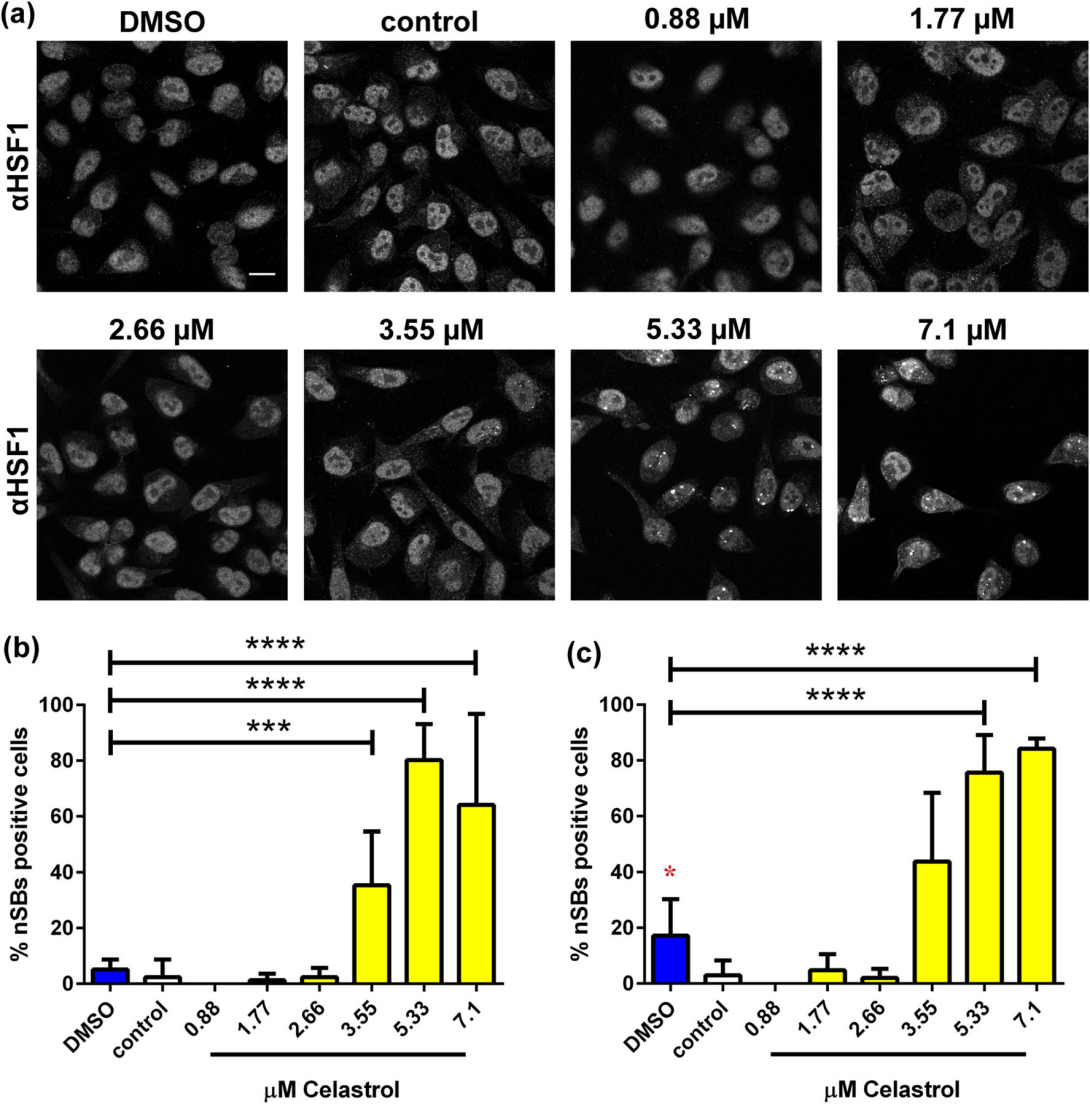
Celastrol induces nSBs formation in Hela cells after 6 h treatment. **a** Confocal images shows that nSBs forms in Hela cells after administration of 3–7 μM of celastrol, lower concentration of celastrol (0.88–2.66 μM) does not clearly induce nSBs formation, scale bar 15 μm. **b** Manually counted nSBs positive Hela cells shows that concentrations over 3.55 μM of celastrol induces significantly more nSBs positive cells than control or DMSO. **c** Computer-based quantification of nSBs positive Hela cells shows that over 5 μM yields significantly higher nSBs positive cell amount than DMSO (0.2% *v*/*v*) treatment. Manually analyzed Hela cells (*n* = 1031), computer-analyzed Hela cells (*n* = 1084), mean ± SEM, *P ≤ 0.05, ***P ≤ 0.001, ****P ≤ 0.0001

It is well studied that celastrol has the ability to induce the transcription of heat shock proteins; however, the ability of celastrol to induce the nSBs formation has remained rather elusive (Trott et al. 2008; Salminen et al. 2010). In this study, we show that celastrol had the ability induce nSBs in similar manner as 42 °C heat shock (Figs. 2 and 3). Furthermore, we determined the formation kinetics of nSBs induced by increasing concentrations of celastrol on human cells, and the results showed that 6 h of treatment with concentrations below 3.55 μM celastrol did not lead to nSBs formation in Hela cells, as no high intensity accumulation of HSF1 could be visually distinguished (Fig. 3a). However, concentrations over 5.33 μM celastrol clearly induced the formation of nSBs in Hela cells, which were detectable as high intensive HSF1 dots in the cell nuclei (Fig. 3a). The highest dose of 7.1 μM also showed nSBs inside the cell nucleus; however, there were fewer cells in those samples, indicating that there might be some toxicity and/or detachment from such a high concentration of celastrol (Fig. 3a).

The quantifications show that Hela cells without any treatment (control) exhibit very low percentage of nSBs positive cells in both manual and computer-based quantification; while vehicle treated (0.2% DMSO) cells exhibit around 20% nSBs positive cells in the computer-based quantification and only 5% in the manual quantification (Fig. 3b, c). Based on this observation, there might be some artifacts in the DMSO-treated samples that the more sensitive computer-based quantifications give higher positive output for, as this is the only group in the whole study that has a significant difference in the two different quantification methods (Fig. 3b, c). More importantly, the quantifications of these immunofluorescence images validate what is seen in Fig. 3; that concentration below 3 μM celastrol does not induce formation of nSBs in Hela cells (Fig. 3b, c). However, a clear increase in nSBs positive Hela cells is observed in the high dose of celastrol-treated cells, suggesting that concentrations ranging from 5.33 to 7.1 μM celastrol are capable of activating the formation of nSBs in Hela cells (Fig. 3). The kinetics for both manually counted and computer-based quantifications is very similar as there is no significant difference between the two drug-treated groups, strengthening the hypothesis that this computerized workflow-based quantification method is reliable for counting nSBs positive cells (Fig. 3b, c).

**Fig. 4:**
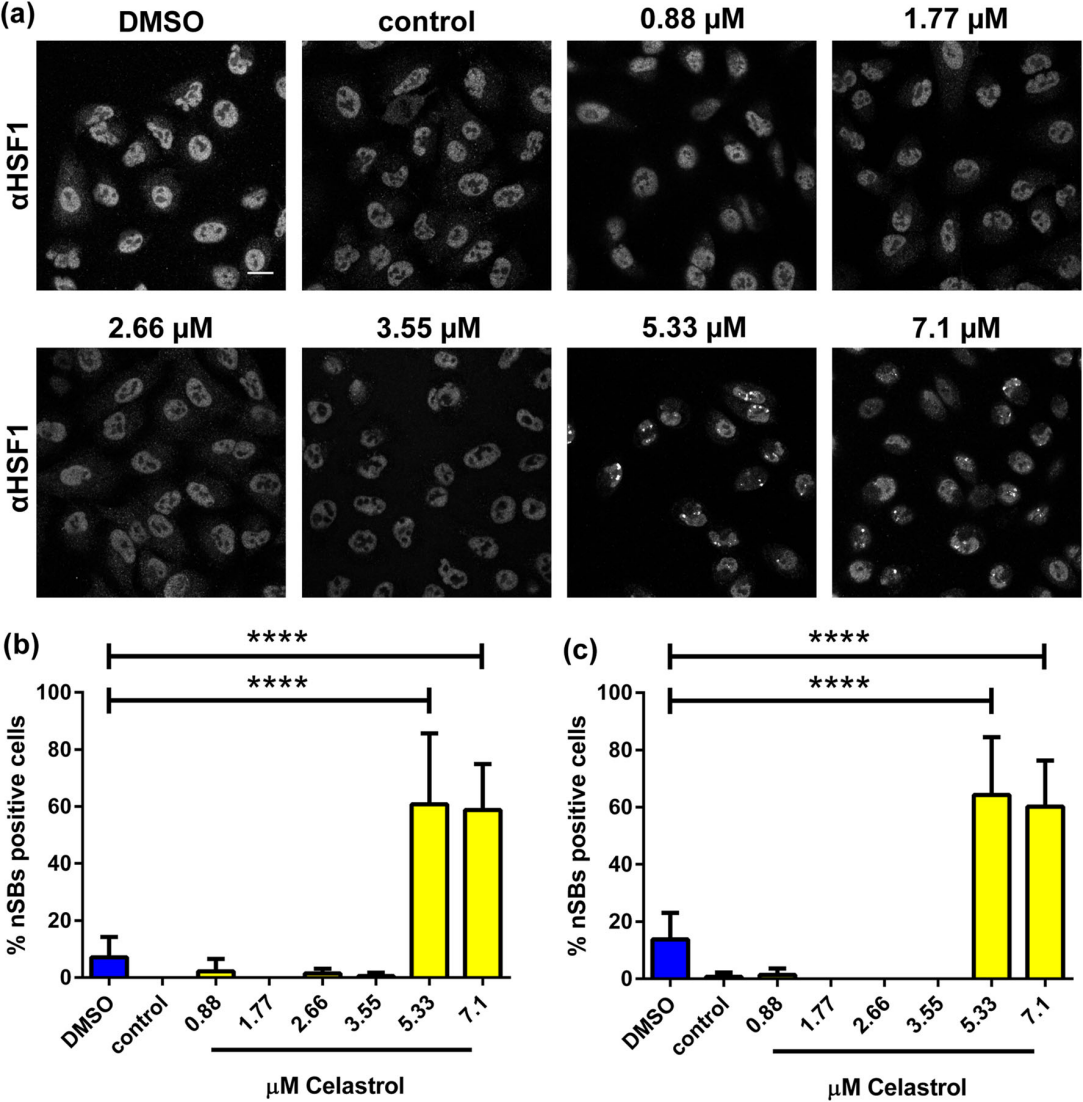
Celastrol induces nSBs formation in A549 cells after 6 h treatment. **a** Confocal images shows that nSBs are clearly formed when administering 5–7 μM of celastrol to A549 cells. Lower concentration of celastrol does not induce nSBs formation in these cells, scale bar 15 μm. **b** Manually counted nSBs positive A549 cells shows that over 5 μM of celastrol is necessary for inducing nSBs in A549 cells. **c** Computer-based quantification of nSBs positive A549 cells shows that over 5 μM celastrol yields significantly higher nSBs positive cell amount than DMSO (0.2% *v*/*v*) treatment. Manually analyzed A549 cells (*n* = 901), computer-analyzed A549 cells (*n* = 904), mean ± SEM, ****P ≤ 0.0001

To further investigate celastrol’s ability to induce formation of nSBs in FR-negative A549 cells (lung epithelial cells), the same concentrations of celastrol as in the experiments with Hela cells was employed for 6 h. The confocal images indicate that concentrations under 3.55 μM celastrol does not lead to formation of nSBs in A549 cells, as no accumulation of HSF1 is detected inside nucleus of these cells (Fig. 4a). Concentrations over 5.33 μM of celastrol were necessary for induction of nSBs in A549 cells, and similarly as the Hela samples, the DMSO-treated A549 cells cause occasionally nSBs-like artifacts in some of the cells (Fig. 4). Results show that these A549 cells need a higher concentration of celastrol for an effective induction of nSBs formation, compared to Hela cells (Figs. 3 and 4). Both the manual- and computer-based quantification shows that concentrations under 3.55 μM celastrol do not induce the formation of nSBs in A549 cells (Fig. 4b, c). However, celastrol concentrations above 5.33 μM clearly induce nSBs formation in A549 cells, proven by both manual- and computer-based quantification (Fig. 4b, c). On the other hand, the highest dose of celastrol (7.1 μM) did not further induce the nSBs formation compared to 5.33 μM in both Hela and A549 cells, and therefore the highest dose was excluded from further experiments, both as free-drug and drug-loaded MSNs (Figs. 3 and 4). The computer-based quantification shows also artifacts in the DMSO-treated A549 cells, detected as 15% of nSBs positive population, compared with the manual estimation that gives less than 5% nSBs positive cells (Fig. 4c). This issue could be minimized with higher celastrol stock solution so that the final concentration of DMSO is lower in all samples or then by changing the solvent used in the stock solution to ethanol for example, as the safety of DMSO have recently been questioned (Galvao et al. 2013). Regardless of the mild DMSO effect, the results shows that the formation of nSBs is caused by celastrol as the number of nSBs positive cells are significantly higher in the high drug concentration treated cells than vehicle treated in both cell lines and both quantification methods (Figs. 3c and 4c). The results show that both heat shock and celastrol have the ability to induce the formation of nSBs in human cells and that quantitative imaging workflows can be used as an accurate method of counting nSBs positive cells that have been subjected with different conditions.

### Characterization of mesoporous silica nanoparticles

**Fig. 5:**
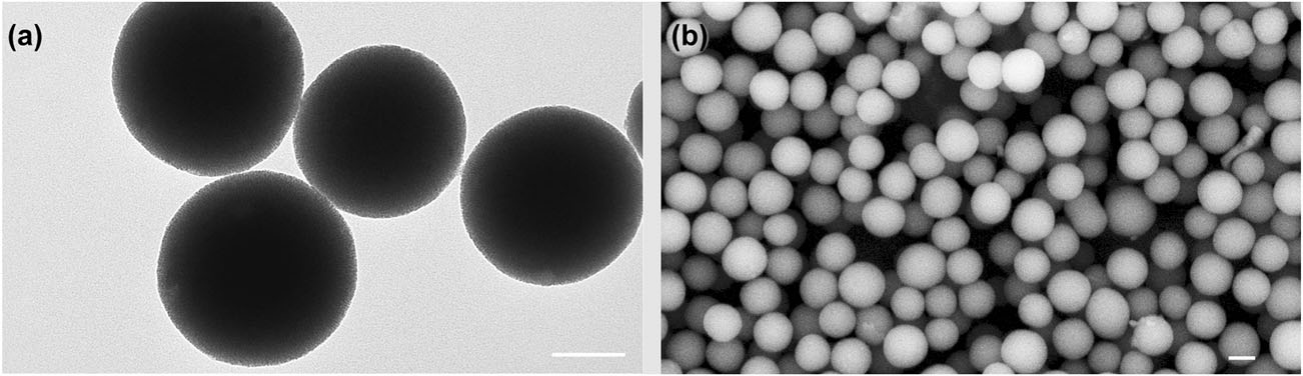
Characterization of the synthetized MSNs. **a** Transmission electron microscopy (TEM) image of the MSNs revealing the porous structure with radially aligned mesopores, scale bar 100 nm. **b** Scanning electron microscopy (SEM) showing multiple MSNs of uniform size with a diameter of 300 nm, scale bar 200 nm

Mesoporous silica nanoparticles (MSNs) were chosen as targeted drug delivery system based on their already established ability to deliver hydrophobic cargo to target cells in vitro with minimal off-target effects (Rosenholm et al. 2009; Niemelä et al. 2015). In this case, folic acid conjugated, poly(ethylene imine), PEI, functionalized MSNs were synthesized and characterized according to previously described methods (Rosenholm et al. 2006, 2008, 2009). Particle size and morphology was determined by scanning and transmission electron microscopy (SEM and TEM), revealing the porous structure of the nanoparticles with a diameter of 300 nm (Fig. 5a, b). The PEI and celastrol amount (in weight % with respect to the whole particle system) was determined by thermogravimetrical analysis (TGA). The PEI amount was estimated to be around 20 wt% and the celastrol loading degree was deduced to be 4.1 wt%. Successful PEI functionalization was further confirmed by change in zeta potential of MSNs from ~ 0 to + 40 mV in HEPES buffer at neutral pH, determined by dynamic light scattering (DLS) technique.

### Cellular uptake of nanoparticles by flow cytometry and confocal microscopy

**Fig. 6:**
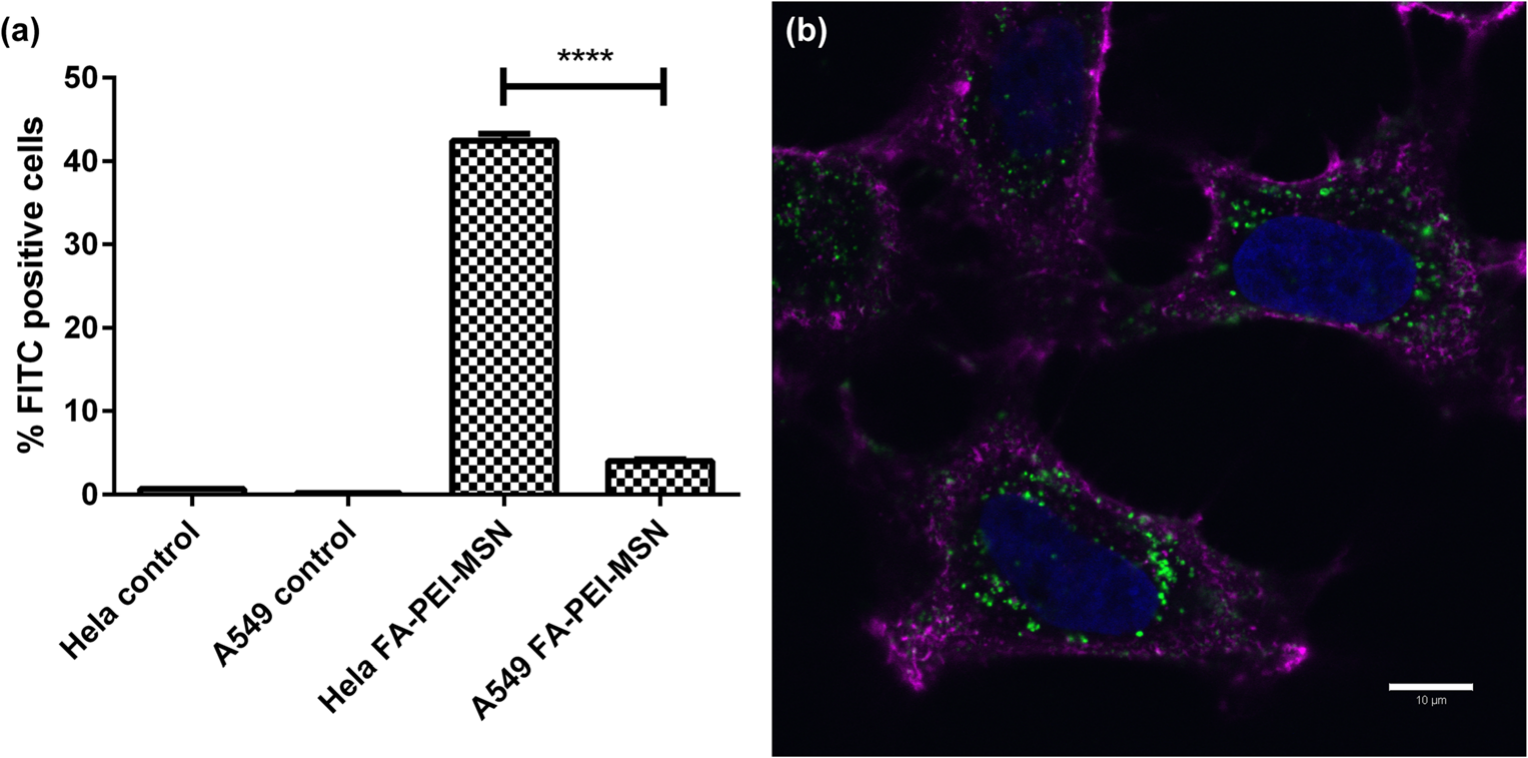
Cellular uptake of FA-PEI-MSNs in cells after 3 h incubation. **a** Flow cytometry quantification showing mean % of FITC positive Hela and A549 cells using 1 μg/ml of nanoparticles or untreated cells (control), independent experiments conducted (*n* ≥ 4), mean ±SEM, ****P ≤ 0.0001. **b** Confocal microscopy images of FA-PEI-MSN endocytosis in Hela cells at 10 μg/ml concentrations, using DAPI nuclear staining (blue), rhodamine-lectin plasma membrane staining (magenta) and particles conjugated with FITC (green), scale bar 10 μm

In order to investigate the cellular uptake of FA-PEI-MSNs in FR-positive Hela cells compared to FR-negative A549 cells, flow cytometry was employed. Cells were subjected to a concentration of 1 μg/ml particles with 3 h incubation; showing that the FR-positive Hela cells had significantly higher particle uptake than FR-negative A549 cells (Fig. 6a). To further validate the specific uptake of these particles in FR-positive cells, confocal microscopy was utilized, showing that these particles were indeed internalized by Hela cells (Fig. 6b). The results demonstrate that FR-positive Hela cells and FR-negative A549 cells work well as model systems for further studying the targeted induction of nSBs formation by celastrol-loaded FA-conjugated nanoparticles for validating our computerized quantification method.

### Celastrol-loaded MSNs induce targeted nSBs formation

**Fig. 7:**
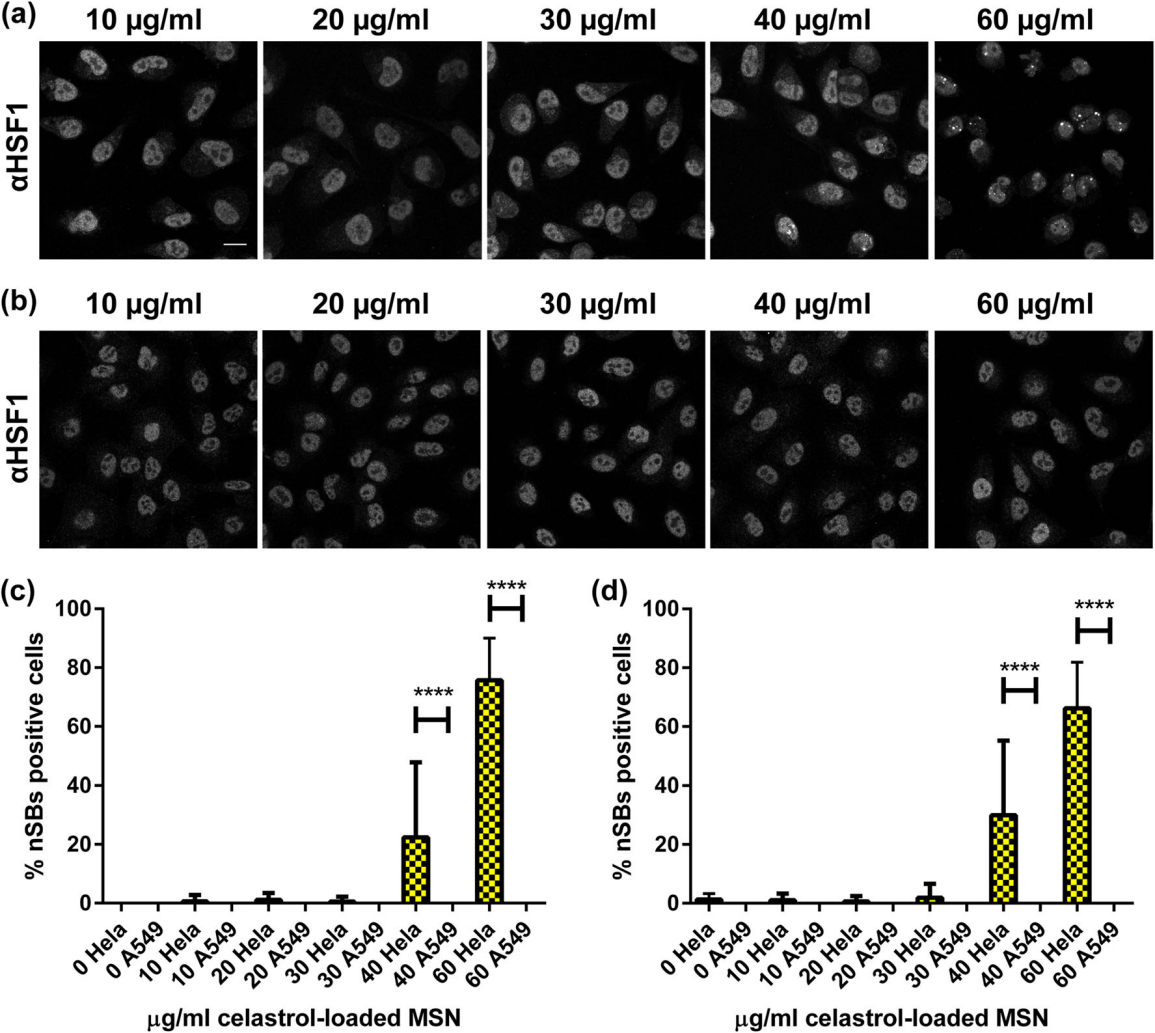
Celastrol-loaded MSNs induce targeted nSBs formation in FR-positive cells. **a** Hela cells treated with celastrol-loaded MSNs shows clear induction of nSBs when administered over 40 μg/ml of drug loaded MSNs. **b** A549-treated cells shows no nSBs formation even the highest dose of 60 μg/ml celastrol-loaded MSNs, scale bar 15 μm. **c** Manually counted nSBs positive cells shows that there is a significant difference between cell lines when treating with 40 and 60 μg/ml of drug-loaded MSN. **d** Computer-based quantification shows similar results where Hela cells show a significant increase in the percentage of stressed cells compared to A549 cells treated with same concentration. Both quantifications methods show that concentration lower than 40 μg/ml does not induce formation of nSBs in Hela or A549 cells. Number of cells analyzed in the manually counted quantification (*n* = 2368), and with the computer-based quantification (*n* = 2436), mean ± SEM, ****P ≤ 0.0001

A major benefit of targeted drug delivery is that the drug concentration in the target tissue is higher compared to the off-target cells, giving rise to a higher efficacy with less side effects, which could be useful in personalized medicine (Singh and Lillard 2009; Rosenholm et al. 2009, 2010; Bae and Park 2011; Zwicke et al. 2012). To examine whether these celastrol-loaded nanoparticles could serve as a targeted inducer of nuclear stress body formation in FR-positive cells, we utilized this computer-based quantification in order to evaluate the target selectivity towards Hela cells and the off-target effect on A549 cells. In comparing the percentage of nSBs positive cells after 6 h treatment of celastrol-loaded nanoparticles in both cell lines, a significant induction of nSBs formation in Hela cells can be detected but not in A549 cells (Fig. 7). Already at 40 μg/ml of celastrol-loaded nanoparticles (around 3.55 μM celastrol), both the manual and computer-based quantifications give a 20% nSBs positive population of Hela cells (Fig. 7c, d). Hela cells treated with 60 μg/ml of celastrol-loaded MSNs (~ 5.33 μM celastrol) had over 60% nSBs positive cells, while A549 cells treated even with the highest dose (60 μg/ml) of drug-loaded particles did not induce any detectable nSBs (Fig. 7c, d). The significant differences in the percentage of nSBs positive cells that can be observed between Hela and A549 cells validates that cells expressing folate receptors are in fact internalizing these celastrol-loaded nanoparticles in higher quantities, which in turn leads to activation and relocalization of the HSF1 to the nucleus that can be detected as nSBs.

Some minor differences between the percentages of nSBs positive cells can be detected with the different quantification methods, as well as variation in the total amount of cells analyzed (Figs. 2, 3, 4, and 7). The most plausible reason for this is because a human is more likely to dismiss a partial cell and might not register a weaker HSF1 signal inside the cell nucleus as an nSBs compared with the more sensitive computer-based quantification. Taken together, the results show that celastrol has the ability to induce the formation of nSBs in human cells by activating and translocating HSF1 to the nucleus, and that it is possible to quantify the immunofluorescence images with our quantification method for detecting and counting HSF1 accumulations. When detecting and measuring nSBs in human cells, the advantages of using computerized workflows compared with manual quantification are accuracy, reliability, and repeatability.

### Downstream effect and cytotoxicity

**Fig. 8:**
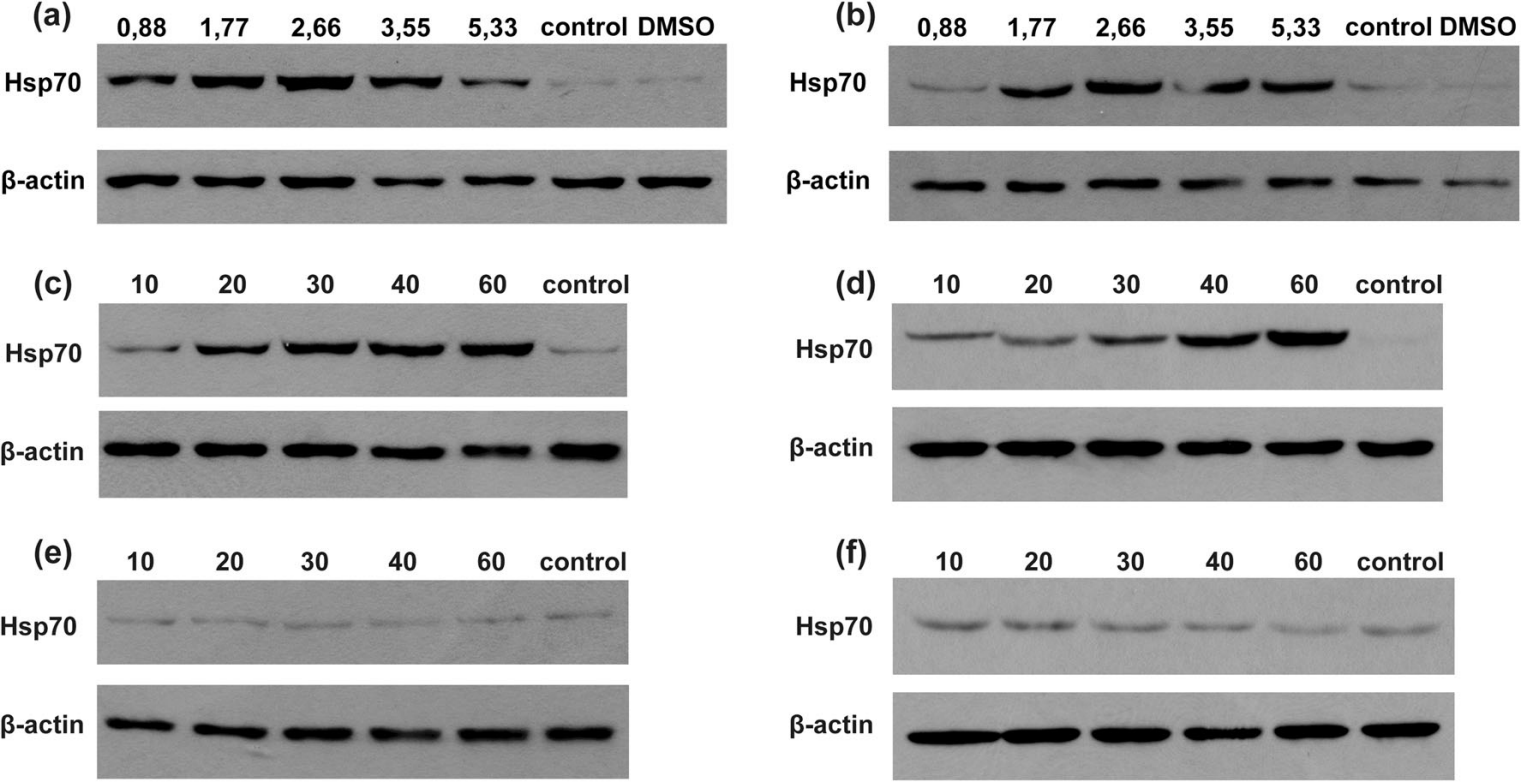
Celastrol-loaded MSNs induces targeted heat shock response in FR-positive cells. **a** Hela and **b** A549 cells treated with increasing concentration (μM) of free celastrol for 6 h shows a stepwise induction of Hsp70 expression. **c** Hela cells treated with increasing concentration (μg/ml) of celastrol-loaded MSNs for 6 h shows an induction of Hsp70 expression in concentrations over 20 μg/ml. **d** A549 cells treated with celastrol-loaded MSNs increases the expression of Hsp70 only at higher concentrations around 40–60 μg/ml. (**c** and d) Both Hela and A549 cells treated with empty MSNs does not increase the expression of Hsp70 compared to untreated cells (control)

To further examine the targeted drug delivery capabilities of these celastrol-loaded MSNs compared to free drug, heat shock protein 70 expression levels were measured by Western blot (WB; Fig. 8). The results show that already a low dose of 20 μg/ml of celastrol-loaded MSNs treated FR-positive Hela cells showing a clear increase of Hsp70 expression, whereas A549 cells lacking FR only shows a clear increase of Hsp70 expression levels when administered a high dose of particles, over 40 μg/ml, indicating that these particles are efficiently delivering their cargo to the target cells (Fig. 8c, d). To validate that the induced heat shock response was not an effect of the particles themselves, corresponding concentrations of empty nanoparticles was administered to Hela and A549 cells for 6 h (Fig. 8e, f). The results show that not even the highest dose (60 μg/ml) of empty nanoparticles did induce the increased expression of the Hsp70 validating that effect was not caused by the folic acid functionalized nanoparticles themselves (Fig. 8e, f).

To confirm that the drug dosage, solvent amount, and particle concentration as well as the time point used in this study does not induce significant toxicity to the selected cell lines; propidium iodide staining by flow cytometry was employed. The action of free celastrol and DMSO vehicle control was first investigated on Hela and A549 cells, and then the toxicity of both empty particles and celastrol-loaded particles was administered and measured on both cell lines. The propidium iodide cytotoxicity assay shows that free celastrol and DMSO only have a minor toxicity in Hela cells and negligible toxicity in A549 cells after 6 h incubation (Supp. Fig. 2). Some toxicity was observed with celastrol-loaded MSN in Hela cells, due to the high uptake of these celastrol-loaded particles in Hela cells, further validating the specificity of these drug-loaded nanoparticles towards FR-positive cells. However, celastrol at higher concentrations has the ability to induce cell death in cancer cells through destabilizing the mitotic spindle and by disrupting topoisomerase II function, thereby leading to DNA fragmentation (Nicoletti et al. 1991; Krysko et al. 2008; Jo et al. 2010; Galluzzi et al. 2012; Niemelä et al. 2015). Regardless of the toxicity of these celastrol-loaded MSN in Hela cells, the apoptotic fraction was still under 15% and should not be a major problem in this work since the focus for this study was to quantify the formation of nSBs, not the toxic effect of celastrol on cancer cells. Furthermore, the empty particles themselves did not show any cytotoxicity measured by flow cytometry, detected as less than 5% apoptotic cells from the total population (Supp. Fig. 2). The empty particles also did not induce the formation of nSBs in the selected cell lines, even if the highest dosage of 60 μg/ml non-loaded MSNs was used for 6 h (Supp. Fig. 3). Taking together, the result shows that these folic acid functionalized celastrol-loaded MSNs works efficiently as targeted drug delivery systems towards folate receptor positive cells with only minimal off-target effects in folate receptor negative cells.

## Discussion

We have investigated the use of a tailor-made BioImageXD-based quantification method for counting and analyzing nuclear stress bodies (nSBs) in human cells, utilizing folic acid (FA) functionalized celastrol-loaded mesoporous silica nanoparticles (MSNs) for targeted induction of the heat shock response in folate receptor (FR) positive cells. First, we validated the computerized quantification method by comparing the nSBs formation kinetics induced by heat shock with the manually based method. The results showed that there is no significant difference between the two methods, which demonstrates that our automated image analysis workflow is accurate, reliable, and versatile for detecting and quantifying nSBs in human cells. Secondly, we utilized celastrol, a pharmacologically active substance that has the ability to activate the heat shock response, in order to validate the specific drug delivery capabilities of these folic acid conjugated MSNs. Thirdly and most importantly, our BioImageXD-based quantification validates that these MSNs indeed have the ability to deliver the poorly soluble drug celastrol specifically to Hela cells expressing folate receptors, with only minimal off-target effect in folate receptor negative A549 cells. This was demonstrated with a statistically significant difference between the nSB positive Hela cell population and the A549 population after administration of these celastrol-loaded MSNs. Furthermore, the downstream effect was studied by Western blot indicating that the celastrol-loaded MSNs increased the heat shock protein levels in Hela cells already at a low dose, and that such low concentrations did not properly induce the stress response in A549 cells.

Overall, this work demonstrates how robust image analysis workflows can be developed and implemented easily in software such as BioImageXD, without the need for programming skills. We also demonstrated the benefits of computerized image analysis compared to manual quantification such as size distribution data; illustrating the usefulness and potential of the additional information obtainable through analyzing 3D data, and that by utilizing batch processing function enabling even high-throughput image processing. Taken together, the results of this study shows that celastrol-loaded MSNs could potentially be beneficial for patients with protein aggregate-associated diseases, such as certain neurodegenerative conditions, by inducing a specific and effective heat shock response in the target tissue. By combining the targeted drug delivery capabilities of these MSNs with our computerized image quantification methods for detecting and characterizing nSBs positive cells, such a theranostic approach could open up possibilities for future automated diagnostics and personalized medicine.

## Electronic supplementary material

ESM 1 (DOCX 1.15 mb)
