# Supplementary material for: Quantitative bioimage analytics enables measurement of targeted cellular stress response induced by celastrol-loaded nanoparticles
Source: Cell Stress Chaperones. 2019 May 11;24(4):735–48. doi: 10.1007/s12192-019-00999-9 (PMC6629742; doi:10.1007/s12192-019-00999-9)
Supplement: Supplementary file 1 — (DOCX 1.15 mb) [file 12192_2019_999_MOESM1_ESM.docx]

‘Supplementary Information

**Quantitative bioimage analytics enables measurement of targeted cellular stress response induced by celastrol-loaded nanoparticles**

Erik Niemelä^a,c^, Diti Desai^b^, Emine Lundsten^a^, Jessica M. Rosenholm^b^, Pasi Kankaanpää^a,*^and John E. Eriksson^a,c,*^

**Characterization of mesoporous silica nanoparticles**


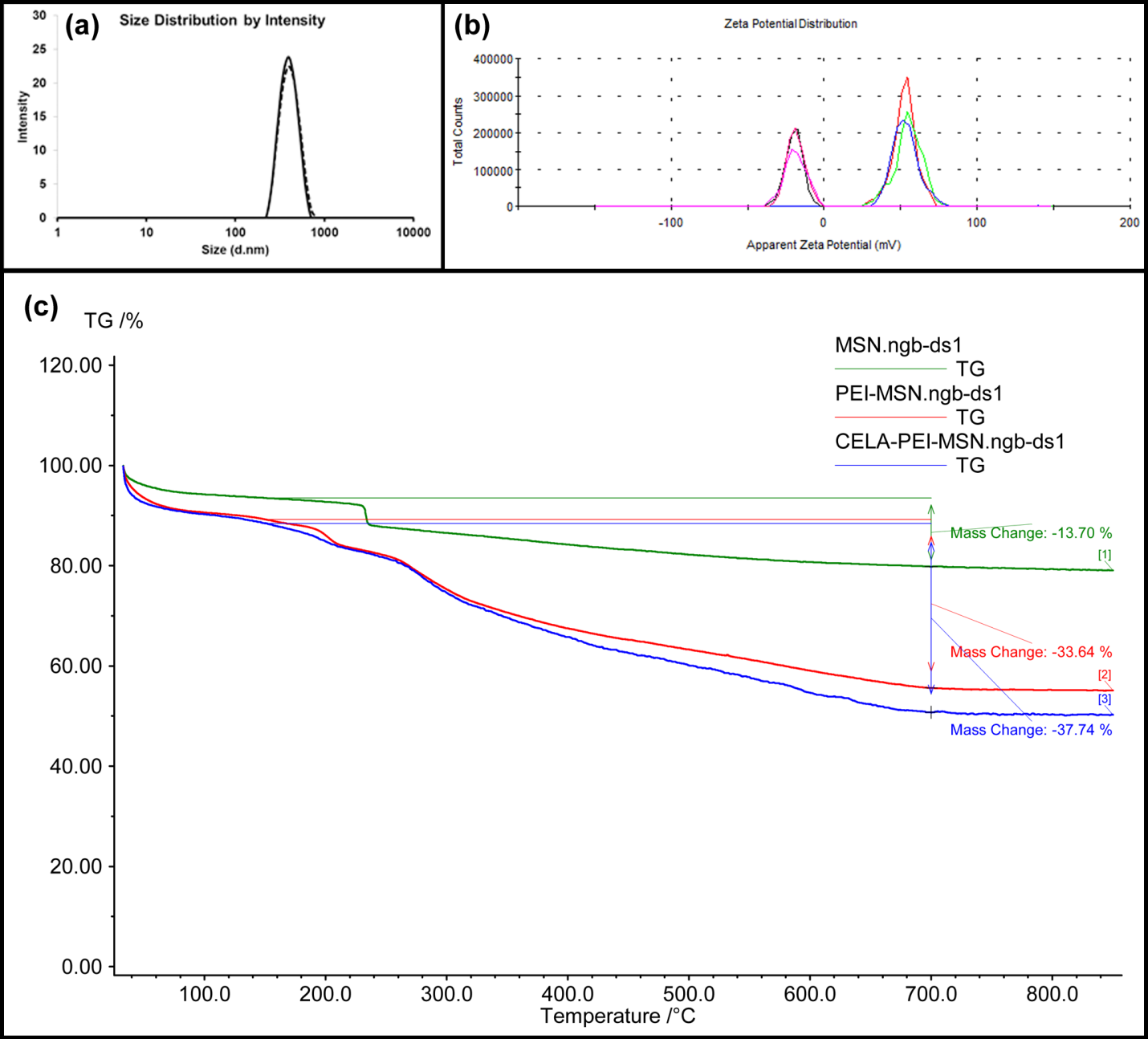


**Supp. Fig. 1. Particle characterization.** a) Hydrodynamic size of MSNs around 300-400 nm determined by dynamic light scattering (DLS) technique (Black: MSN-PEI-FR), b) Thermogravimetric analysis (TGA) show that the PEI amount in the MSN is around 20-weight % and that the deduced amount of celastrol loaded to be 4.1 weight % (Blue: MSN; Black: MSN-PEI).

The hydrodynamic size of the produced MSNs was determined by dynamic light scattering (DLS) technique (Supp. Fig. 1a), and found to be around 300-400 nm when measured in physiological buffer (HEPES buffer, pH 7.2). DLS provides information regarding particle size in the solution, thus confirming full redispersibility of particles in HEPES buffer. The PEI amount (in weight % with respect to the whole particle system), as determined by thermogravimetrical analysis (TGA), was around 20-weight % (Supp. Fig. 1c). Successful PEI functionalization was further confirmed by change in zeta potential from of MSNs ~ 0 mV to + 40 mV, as seen in Supp. Fig. 1b. The amount of celastrol loaded to the MSNs was deduced from TGA and found to be 4.1 weight % for PEI-MSNs (Supp. Fig. 1c).

**Cytotoxicity measurement by flow cytometry**

One of the hallmarks of apoptosis is that the DNA gets fragmented; which can be exploited by flow cytometry when utilizing propidium iodide (PI) staining that bind to fragmented DNA, and when this binding occurs the excitation wavelength of PI changes that can be detected.^1-3^ Hela and A549 cells were seeded in a 12-well culture plate and incubated with free celastrol, celastrol-loaded nanoparticles or empty nanoparticles (without celastrol) or vehicle (0.2% v/v DMSO) for 6 hours (Supp. Fig. 2). After incubation, cell medium was collected and the cells were washed, and then harvested by incubation with trypsin-EDTA for 5 minutes at 37 °C. Samples were centrifuged for 5 minutes and then washed with PBS and re-suspended in 400 µl of lysis buffer (0.3% Triton X-100, 0.05 mg/ml PI, 40mM sodium citrate and 1 mg/ml RNase A) for 10 minutes at 37 °C in order to obtain the nuclear fraction. Cells were immediately analyzed by a BD FACSCalibur flow cytometer (FL-3, BD Pharmingen, San Diego, California, USA) at 488 nm excitation and 670 nm emission with 10000 cells analyzed per sample. The percentage of apoptotic cells defined as sub-G1 phase was analyzed using BD CellQuest Pro™ software (BD Pharmingen, San Diego, CA, United States).^44^


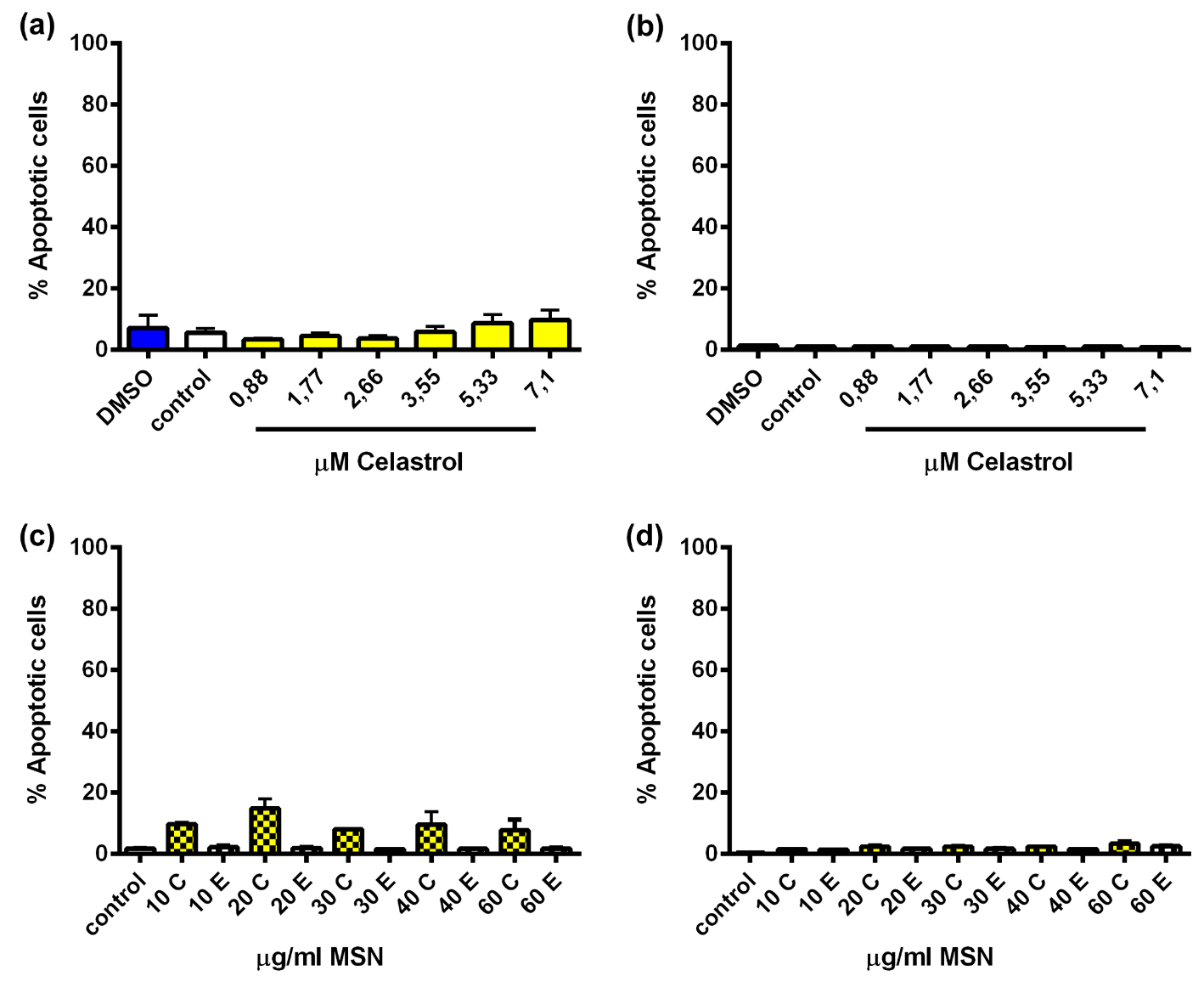


**Supp. Fig. 2.** Flow cytometry toxicity measurement of free celastrol or celastrol-loaded MSNs in Hela and A549 cell lines. (a) Hela cells incubated with celastrol or DMSO (0.2% v/v) for 6 hours shows only minor toxicity even at high concentration detected using propidium iodide (PI) staining. (b) A549 cells treated with celastrol shows neglectable toxicity even at 7.1 µM concentrations. The toxicity of empty MSNs (without celastrol denoted as E) and celastrol-loaded MSNs (denoted as C) was also measured. (c) Hela cells incubated for 6 hours with drug loaded MSNs shows minor toxicity compared to empty particles probably due to the high uptake of the celastrol-loaded particles that induces mitotic catastrophe in fast dividing cells. (d) A549 cells treated with drug loaded or empty particles did not show any toxicity not even in the high dose. Error bars represents ±SEM (n=2).

**Non-drug loaded MSNs do not induce the heat shock response**

In order to rule out that the formation of nuclear stress bodies (nSBs) is not due to the nanoparticles themselves. Non-drug loaded (empty) MSNs ability to induce formation of nSBs in both cell lines were investigated by confocal microscopy, using the same time points and concentrations as with the celastrol-loaded MSNs experiments for detecting the induction of the heat shock response. The confocal images shows that there is no formation of nSBs in either cell lines, even when administered up to 60 µg/ml of empty nanoparticles (Supp. Fig. 3.).


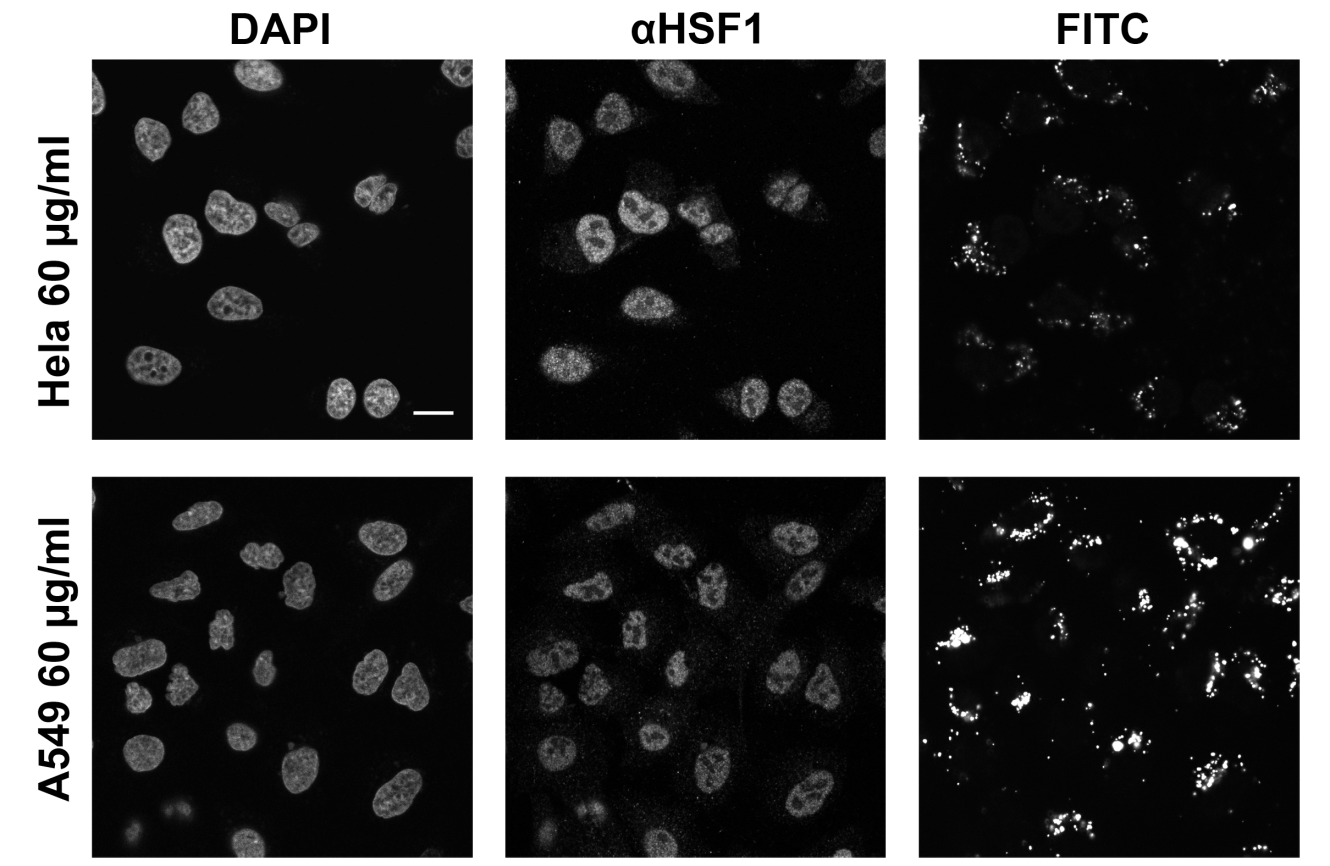


**Supp. Fig. 3. Non-loaded MSNs do not induce nSBs formation in human cells.** Hela and A549 cells treated with 60 µg/ml non-loaded MSNs for 6 hours do not induce formation of nSBs, validating that the induction of nSBs are not an effect of the MSNs alone. Images taken with 63x magnification with a scale bar of 15 µm.

**References**

Nicoletti I, Magliorati G, Pagliacci M, Grignani F,Riccardi C (1991) A rapid and simple method for measuring thymocyte apoptosis by propidium iodide staining and flow cytometry. J Immunol Methods 139.

Krysko DV, Vanden Berghe T, D'Herde K, Vandenabeele P (2008) Apoptosis and necrosis: detection, discrimination and phagocytosis. Methods 44(3):205-21. doi: 10.1016/j.ymeth.2007.12.001.

Galluzzi L, Vitale I, Abrams JM et al (2012) Molecular definitions of cell death subroutines: recommendations of the Nomenclature Committee on Cell Death 2012. Cell Death Differ 19(1):107-20. doi: 10.1038/cdd.2011.96.
